# A mentored hands-on training model for scaling up implementation and intervention research in India: “connecting the dots”

**DOI:** 10.1186/s12961-023-00980-0

**Published:** 2023-05-02

**Authors:** Ramdas Ransing, Mary Hawk, Margaret McDonald, Jacquelyn Jones, Triptish Bhatia, Vijay Verma, Gyan D. Shah, Jaspreet Brar, James Erin Egan, Prasad Konsale, Jasmine Kaur, Ravinder Singh, Harpreet Singh, R. S. Dhaliwal, Joel Wood, Vishwajit Nimgaonkar, Smita Deshpande, Soumya Swaminathan

**Affiliations:** 1 Department of Psychiatry, BKL Walwalkar Rural Medical College, Sawarde, Ratnagiri, Maharashtra 415606 India; 2 Department of Behavioral and Community Health Sciences, University of Pittsburgh School of Public Health, Pittsburgh, PA 15261 United States of America; 3 Department of Epidemiology, University of Pittsburgh School of Public Health, Pittsburgh, United States of America; 4 Department of Psychiatry, University of Pittsburgh School of Medicine, Pittsburgh, PA 15213 United States of America; 5 National Coordinating Unit ICMR for NMHP Projects, Department of Psychiatry, Centre of Excellence in Mental Health, ABVIMS, Dr Ram Manohar Lohia Hospital, New Delhi, 110001 India; 6 Department of Psychiatry, Western Psychiatric Institute and Clinic, Community Care Behavioral Health Organization, University of Pittsburgh School of Medicine, Pittsburgh, PA 15213 United States of America; 7 University of Pittsburgh School of Medicine, University of Pittsburgh Swanson School of Engineering, VA Pittsburgh Healthcare System, Pittsburgh, PA 15213 United States of America; 8 Indian Council of Medical Research, Ansari Nagar, New Delhi, 110029 India; 9 Psychiatry and Human Genetics, University of Pittsburgh School of Medicine and Graduate School of Public Health, Pittsburgh, PA United States of America; 10 Department of Psychiatry, Centre of Excellence in Mental Health, ABVIMS - Dr. Ram Manohar Lohia Hospital, Bangabandhu Sheikh Mujib Road, New Delhi, 110001 India; 11 World Health Organization, Geneva, Switzerland

**Keywords:** Grantathon, Mental health, Research mentoring, Capacity-building

## Abstract

Despite the high burden of mental disorders in low- and middle-income countries (LMICs), less than 25% of those in need have access to appropriate services, in part due to a scarcity of locally relevant, evidence-based interventions and models of care. To address this gap, researchers from India and the United States and the Indian Council of Medical Research (ICMR) collaboratively developed a “Grantathon” model to provide mentored research training to 24 new principal investigators (PIs). This included a week-long didactic training, a customized web-based data entry/analysis system and a National Coordination Unit (NCU) to support PIs and track process objectives. Outcome objectives were assessed via scholarly output including publications, awards received and subsequent grants that were leveraged. Multiple mentorship strategies including collaborative problem-solving approaches were used to foster single-centre and multicentre research. Flexible, approachable and engaged support from mentors helped PIs overcome research barriers, and the NCU addressed local policy and day-to-day challenges through informal monthly review meetings. Bi-annual formal review presentations by all PIs continued through the COVID-19 pandemic, enabling interim results reporting and scientific review, also serving to reinforce accountability. To date, more than 33 publications, 47 scientific presentations, 12 awards, two measurement tools, five intervention manuals and eight research grants have been generated in an open-access environment. The Grantathon is a successful model for building research capacity and improving mental health research in India that could be adopted for use in other LMICs.

**Supplementary Information:**

The online version contains supplementary material available at 10.1186/s12961-023-00980-0.

## Background

Despite the high burden of mental disorders in low- and middle-income countries (LMICs), only about 10–25% of people with mental illness are able to access needed services [1–3], in part due to a scarcity of locally relevant, evidence-based tools, interventions and models of care [4]. Low numbers of providers, inadequate training and a paucity of funds for implementation research and the conduct of the research itself are contributing factors [1, 5, 6], resulting in insufficient services, despite India’s National Mental Health Programme (NMHP). There is a growing need to strengthen the pool of mental health researchers in intervention research to improve the delivery and assessment of care under the NMHP.

**Fig. 1:**
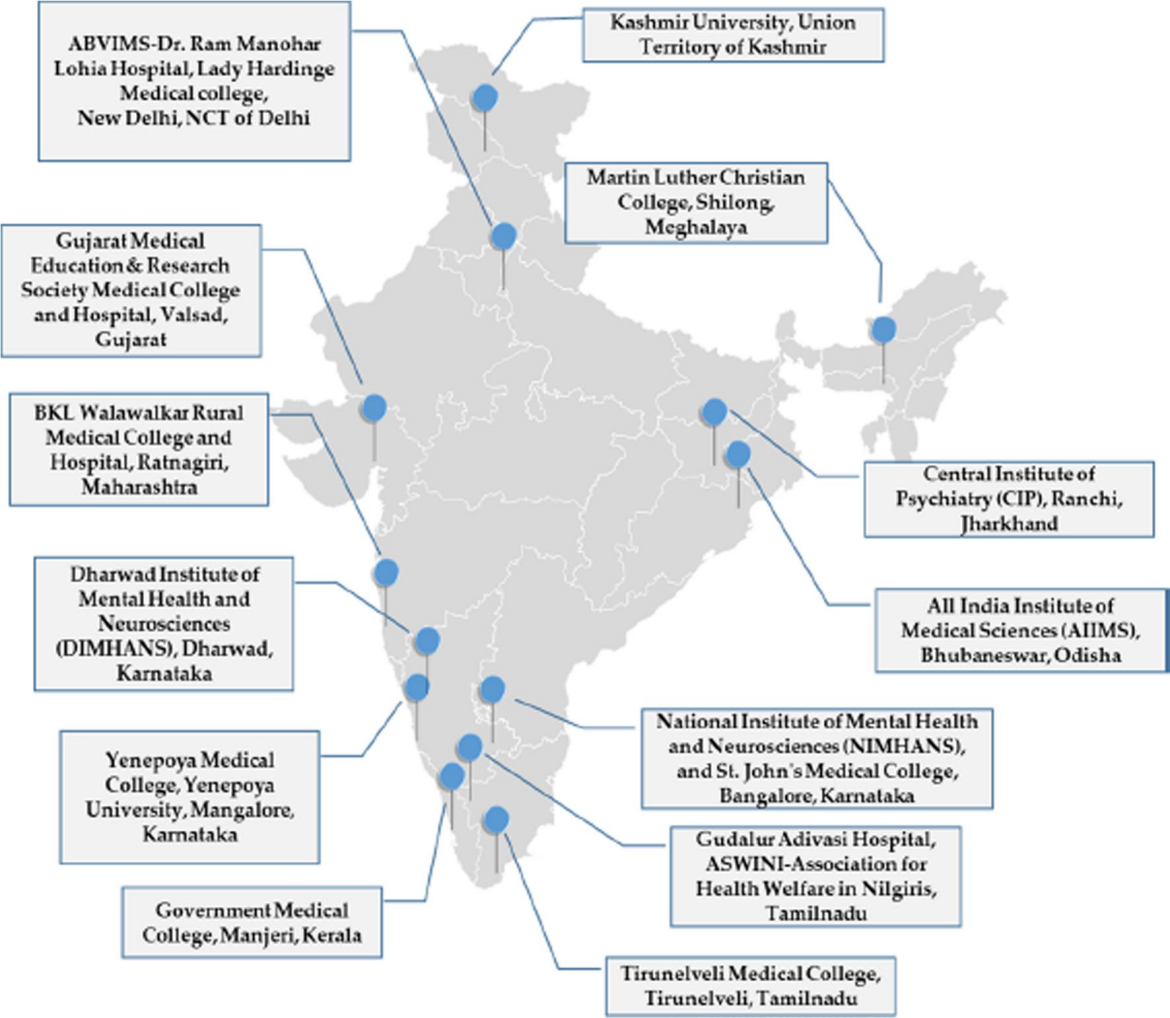
Research project sites across the country. Jammu and Kashmir state has been divided into two union territories (Jammu and Kashmir, Ladakh)

To address this issue, the Indian Council of Medical Research (ICMR), in conjunction with Indian and United States academic researchers, developed a capacity-building “Grantathon” model for training new investigators in intervention research, with funding from the United States National Institutes of Health [7–9]. The Grantathon trained new researchers, mentored them and helped to develop innovative, interdisciplinary and collaborative projects that could be nationally adopted under the NMHP. Twenty-four principal investigators (PIs) from institutions across India participated in the 2016 Grantathon [7]. ICMR subsequently funded 12 single-centre or multicentre projects, which were implemented across study sites in India (Fig. 1) beginning in October 2018 [9]. This paper describes subsequent mentoring methods, challenges and solutions, and outcomes demonstrated by this Grantathon-funded set of studies.

## Methods

The rationale and processes we used to develop the Grantathon model are fully described elsewhere [7]. Briefly, PIs were assisted through all phases of study conceptualization, development and implementation by mentors assigned during the 2016 Grantathon and supported by the national coordinating unit (NCU) located at Dr. Ram Manohar Lohia Hospital (RMLH), New Delhi [7, 9]. The data management unit (DMU), located at ICMR headquarters in New Delhi and led by a senior scientist, actively collaborated with the NCU and PIs to develop data entry, quality control and data management through i-MANN (ICMR-Mental Health Assessment National Network), a new database developed specifically for these projects [10]. Project-specific standard operating procedures were developed to detail activities, responsibilities and actions. These were signed by all study partners and used to track implementation and monitor progress [9]. DMU team members supported PIs in resolving administrative issues relating to research and software development and provided day-to-day assistance with i-MANN.

Each PI or group of PIs was assigned one or more mentors who monitored progress and advised on project implementation. As projects were implemented across India and trainees were supported by international mentors, virtual platforms (e.g. WhatsApp, videoconferencing) were used for training, mentoring and monitoring. Mentors also visited sites to guide PIs, and PIs of multicentric studies visited each other’s sites to learn and teach. In-person review meetings were organized semiannually to discuss the progress of individual projects. Challenges and difficulties experienced by PIs were discussed, and practical solutions, support and guidance were provided. Issues addressed included those related to research methods and implementation, participant retention strategies and grant management. Mentoring topics also included emerging scientific queries, qualitative and quantitative analysis, and strategies for avoiding pitfalls related to methodology, ethics and data entry. Mentors assessed the need for additional training and subsequently organized workshops during review meetings addressing ethics, qualitative methods, dissemination of findings and other methods-related topics. PIs were also encouraged to attend courses, seminars and conferences, and to publish their research. Assistance was provided for developing manuals, training courses and publications, with international and NCU mentors actively involved in iterative processes of manuscript and proposal development and submission. New opportunities for funding, training and dissemination of results via conferences were also shared.

The NCU monitored PIs’ progress through monthly virtual meetings or phone calls, augmenting one-on-one and group mentorship provided by faculty at the University of Pittsburgh and other senior researchers at participating institutions within India. Review meetings were also conducted by the NCU every 6 months to monitor progress, encourage mutual learning and provide additional training to PIs on research methods. While the first two meetings took place in person in India, subsequent meetings were held via videoconferencing due to COVID-19 travel restrictions. In these meetings, mentors and experts not directly associated with the projects critically assessed progress and provided feedback and constructive suggestions to advance the work. These regular updates aimed to maximize productivity in research, publications and presentations. Regular meetings with NCU staff, international mentors and one another helped build collaborative research relationships.

Evaluation of the Grantathon projects was challenging, especially given the wide range of topics, populations and study designs. To ensure adequate flexibility to review these diverse projects and provide critical feedback, a brief evaluation plan was developed that was sufficiently broad to assess each of the projects and the Grantathon as a whole. Process objectives tracked by the NCU included participant recruitment rates and rigour in data collection. Outcome objectives were assessed via scholarly output including publications, awards received and leveraging of additional grants.

## Results

Figure 1 shows the regions of India where projects were implemented, and a brief description of the projects funded under the Grantathon model is provided in Table 1. All projects were based broadly on the thematic foci of the NMHP: reducing the treatment gap and developing integrative and sustainable models, improving the accessibility and availability of mental health services and promoting community participation in mental healthcare. Strengthening the capacity of government agencies to initiate and sustain such efforts in real settings (e.g. project ECHO [Extension for Community Healthcare Outcomes]) was emphasized.

**Table 1:** Salient features of individual research projects funded by ICMR and mentored by CFRT

Sr.No.	Full name and short title of ongoing projects	Research domains	Number of mentors/PIs	Problems addressed by the model	Proposed strategy to address	Stakeholders	Linkages to NMHP
1.	A multicentric randomized controlled trial to assess the effectiveness of screening and a brief nurse-delivered intervention for depression in pregnancy (BIND-P)	Perinatal depression, interventional, multicentric (Maharashtra, Karnataka, New Delhi)	2/4	Recognizing depressive symptoms in antenatal care, improving accessibility to perinatal mental health services	Stepped care model involving primary healthcare workers	• Primary healthcare workers • Nurses • Government (state and union) • Specialists in the fields	Integrative, collaborative models, early interventions
2.	Alcohol use among adolescent tribals in three corners of India: prevalence and pilot intervention studies	Tribal mental health, addiction, interventional, multicentric (Tamil Nadu, Gujarat, Meghalaya)	1/4	Alcohol use/dependence in tribal adolescents and improving their life skills	Survey followed by life skills workshops, follow-ups	• Adolescents and their families • Tribal communities • Healthcare workers in tribal area	Early detection, intervention through training in life skills
3.	A multicentric randomized controlled trial to evaluate the efficacy of telephone-based psychosocial interventions on future suicide risk in suicide attempters (TOPS)	Telepsychiatry and suicide prevention, interventional study, multicentric	2/4	Telephone intervention to prevent recurrence of a suicide attempt	Identification and follow-up of persons reporting to the hospital with a suicide attempt	• Persons attempting suicide and their families • Healthcare workers in the field • Specialists • Suicide prevention policy-makers	Suicide prevention through cost-effective outreach models
4.	Multicentric randomized controlled trial comparing effectiveness of fluoxetine and mindfulness in primary care (DIAMAND)	Noncommunicable disease and primary care physician, interventional, multicentric	2/5	Mild to moderate depression among persons with diabetes	Treating mild to moderate depression among persons with diabetes with medication, mindfulness training in a randomized controlled trial format, detection by their primary care physicians	• Persons with diabetes • Primary care physicians • Clinical psychologists, • DMHP policy-makers • Psychiatrists	Diabetes is highly prevalent in India, depression is a common problem in diabetes
5.	Implementation and evaluation of the NIMHANS-ECHO blended training programme for the DMHP workforce in a rural South Indian district of Karnataka State (NIMHANS-ECHO-1)	Tele-mentoring of DMHP personnel, interventional, single-site	4/1	Identification and primary management of mental disorders at the primary care centre	Tele-mentoring	• Auxiliary nurse midwives (ANMs) • Accredited social health activist (ASHA) workers • Medical officers of primary health centres	Management of mental disorders in primary healthcare
6.	Effectiveness of addition of virtual NIMHANS ECHO tele-mentoring model for skilled capacity-building in providing quality care in alcohol use disorders (AUDs) by existing staffs of the DMHP districts of Karnataka (NIMHANS-ECHO-2)	Tele-mentoring, alcohol use and dependence, single-site	4/1	Identifying and managing alcohol use disorders at the primary level	Tele-mentoring of healthcare workers	• Primary health centre medical officers	Identification and management of alcohol use disorders in primary care
7.	Community-based intervention on mental health in Kashmir	Community-based intervention, single-site	3/1	Stress and coping issues due to environmental circumstances	Survey for identifying vulnerable persons, offering psychological counselling	• Counselling psychologists	Due to stressful environment, mild mental disorders are believed to be common, and require intervention
8.	Effectiveness of community-based rehabilitation delivered by ASHAs for persons with severe mental illness in a rural community in Karnataka: a randomized controlled comparison with specialist-delivered care	Community-based rehabilitation, primary healthcare workers, single-centre	2/1	Rehabilitation of persons with schizophrenia	Home-based follow-up by lay primary care workers	• ASHAs	Recovery and prevention of relapse in severe mental illness
9.	Development and validation of the screening version of Indian Scale for Assessment of Autism	Scale development, single-centre	4/1	Screening children and adults for symptoms of autism	Community screening of children	• Minimally trained community workers	Early screening and referral of at-risk children
10.	Psychological intervention by videoconference for vulnerable family members of farmers who have committed suicide	Telepsychiatry for grief and at-risk population, single-site	1/1	Depression among family members of farmers who died by suicide	Screening families and offering counselling	• Psychologists	Farmers are an at-risk group for suicide, and their families need support
11.	Outcome of services at the community extension clinics for patients with common mental disorders (CMDs): a client-centred approach	Community-based mental health services, single-centre	4/1	Psychiatric facility	Screening and discussion with patients using these facilities	• Patients	Improving service delivery
12.	Development of a community-level module for physical illnesses in patients with psychiatric illness	Development of a community-level module for physical illness with psychiatric illness, single-site	1/1	Community screening for persons with mental illness	Persons with mental illness receive physical examination and tests	• Community workers	Reducing burden of physical comorbidities in persons with mental illnesses
13.	Evaluation of District Mental Health Programme (DMHP) psychiatric services to the severely mentally ill in their old age	Evaluation of district mental health services, single-centre	4/1	Cluster-sampling followed by group discussions	Elderly who suffer from mental health issues are identified and offered care	• Primary healthcare workers • Public health specialists	Improving mental healthcare for the elderly

*ANM* auxiliary nurse midwife, *ASHA* accredited social health activist, *CFRT* cross-fertilized research training, *DMHP* District Mental Health Programme, *ECHO* Extension for Community Healthcare Outcomes, *NIMHANS* National Institute of Mental Health and Neuro-Sciences

### Process objectives

Given that a major goal of the Grantathon model was to build research capacity throughout India, it was critical to create infrastructure enabling collection of information to monitor study implementation in terms of participant recruitment, intervention and reach. The COVID-19 pandemic underscores the importance of reliable data to drive intervention and policy decisions. All rating scales used by the PIs were digitized (*n* = 77) and added to i-MANN for ease of access. PIs were required to regularly enter their data online into this database, ensuring adherence to recruitment timelines and achievement of time-bound implementation milestones. Site-specific data entries were assessed during the regular review meetings. i-MANN proved to be a significant resource for monitoring and evaluation. Moreover, the dashboards provided to PIs were routinely used for reviewing the work of project staff at each site. i-MANN was developed using modular architecture and open-source technologies with a standardized DHIS2-based platform and can be easily made available to all the researchers working on mental health nationwide. Once sufficient data are collected on various aspects of mental health, they can be used for modelling hotspots, conducting predictive analysis and devising interventions to promote awareness and improve quality of life for people with mental illness. Three years post-Grantathon, the database has collected 23,468 records on 10,489 unique study participants across India. Overall, the projects achieved their targets in terms of quality, project milestones and number of participants recruited. De-identified data from i-MANN can be made available for use by accredited researchers with appropriate permission from PIs and ICMR.

### Outcome objectives

All research protocols were published in a supplement of the *Indian Journal of Psychological Medicine*. The supplement was, in itself, an important accomplishment as the first protocol supplement published in India. Making these protocols available enables other researchers to replicate these intervention models [11]. Findings from many projects are regularly presented at conferences and scientific meetings. Total outcomes resulting from the Grantathon to date are presented in Table 2 and include the number of manuscripts published (*n* = 30) or accepted for publication (*n* = 3 at the time of this writing), dissemination of findings in conferences or scientific meetings (*n* = 47), development of manuals (*n* = 5) and new scales/questionnaires (*n* = 3), symposia organized at conferences (*n* = 3), new research grants (*n* = 8) (Additional file 1: Table S1) and scholarly (*n* = 12) or scientific (*n* = 10) awards received by PIs. The number of staff members trained and broad descriptions of activities conducted under each project are shown in Table 2.

**Table 2:** Three-year outcome of individual projects

Short title of ongoing project	Staff trained	Measurable scientific outcome (*n*)^a^	Contribution of NMHP
A multicentric randomized controlled trial to assess the effectiveness of screening and a brief nurse-delivered intervention for depression in pregnancy (BIND-P)	JRFs: 12 Nurses: 75 Postgraduates (psychiatry and allied): 18 Data entry operators: 1 Nursing students: 143	Publications: 6 Presentations: 15 Awards: 8 Manuals: 4 New research grants: 2	• Developed a proof-of-concept model for perinatal depression • Intervention guide and training manual in three Indian languages (Marathi, Hindi, Kannada) • Findings have NMHP policy implications such as incorporating mandatory depression screening for perinatal women, delivery of intervention through nonspecialist health workers in primary care settings • Study findings were already presented to the programme coordinator of the District Mental Health Programme of Dakshina Kannada (Karnataka) • Department of public health (Maharashtra) to implement the intervention
Alcohol use among adolescent tribals in three corners of India: prevalence and pilot interventional studies	JRFs and project staff: 12 Adolescents trained in LSEM: 25	Publications: 1 Presentations: 1	• Development of preventive and supportive mental healthcare model for tribal youth
A multicentric randomized controlled trial to evaluate the efficacy of telephone-based psychosocial interventions on future suicide risk in suicide attempters (TOPS)	JRFs: 9 Data entry operators: 1 PhD scholars: 1	Publications: 4 Presentations: 12 Awards: 1 Manuals: 1 New research grants: 4	• Development of telephone-based interventions and model of care to prevent suicide in high-risk individuals • Training of nonspecialist mental health workers in delivery of psychosocial interventions to prevent suicide in high-risk individuals
A multicentric randomized controlled trial comparing effectiveness of fluoxetine and mindfulness in primary care: protocol for DIAbetes Mellitus ANd Depression (DIAMAND) study	JRFs: 12 Primary healthcare professionals: 49	Publications: 3 Presentations: 1 Awards: 2	• Development of a brief manual for primary healthcare physicians to detect and manage depression in primary care settings (Hindi, English) • Development of training module for primary healthcare workers in identifying and treating depression in primary care settings • Development of a virtual protocol during the COVID-19 pandemic
Implementation and evaluation of the NIMHANS-ECHO blended training programme for the DMHP workforce in a rural South Indian district of Karnataka State (NIMHANS-ECHO-1)	JRFs: 4 ANM: 6 ASHAs: 86 Medical officers: 8 Multipurpose health workers: 3	Publications: 3 Presentations: 5	• Establishing the feasibility of a digital technology-based model for training of grassroots health workers in mental health • Model for screening for mental illness by grassroots-level health workers • Development of a mental health screening and counselling tool for field-level workers of India (MERIT)
Effectiveness of addition of virtual NIMHANS ECHO tele-mentoring model for skilled capacity-building in providing quality care in alcohol use disorders (AUDs) by existing staffs of the DMHP districts of Karnataka (NIMHANS-ECHO-2)	JRFs: 2	Publications: 1 Presentations: 2	• Establishing the feasibility of a tele-mentoring model for training DMHP staff for AUDs • Model for improving the quality of care for AUDs
Community-based intervention on mental health in Kashmir	JRFs: 2 SRFs: 1 Community health workers: 30	Publications: 3 Presentations: 3 New research grants: 1	• Model for community-based awareness, screening and intervention for mental illness • Training of the community health workers for the sustainable intervention at the community level
Effectiveness of community-based rehabilitation delivered by ASHAs for persons with severe mental illness in a rural community in Karnataka: a randomized controlled comparison with specialist-delivered care	JRFs: 1 ASHAs: 101	Publications: 5 Presentations: 3 Awards: 1	• Development of cost-effective, up-scalable and replicable community-based rehabilitation for persons with severe mental illness through ASHA workers in resource-poor settings
Development and validation of the screening version of the Indian Scale for Assessment of Autism	JRFs: 4	Publications: 2 Presentations: 1 New research grants: 1	• Development of screening version of the Indian Scale for Assessment of Autism
Psychological intervention by videoconference for vulnerable family members of farmers who have committed suicide	JRFs: 2	Publications: 1 Presentations: 1	• Model for delivery of psychosocial intervention for vulnerable family members using videoconferencing
The outcome of services at the community extension clinics for patients with common mental disorders (CMDs): a client-centred approach	JRFs: 2	Publications: 2 Presentations: 1	• Development of community-based services for patients with CMDs
Development of a community-level module for physical illnesses in patients with psychiatric illness	JRFs: 2	Publications: 1 Presentations: 1	• Delivery of community-level intervention for physical illnesses in patients with psychiatric illness
Evaluation of District Mental Health Programme (DMHP) psychiatric services to the severely mentally ill in their old age	ASHAs: 1310 JRFs: 6	Publications: 1 Presentations: 1	• Screening for elderly patients with severe mental illness (SMI) • Development of community-based tool to identify elderly patients with SMI and interventions to improve the access to care under DMHP. The service and barriers to the access of care among the elderly patients with SMI were assessed from a sample of 917 participants • A tool was developed which would be helpful for NMHP to quickly identify the elderly SMI from communities • An intervention programme is being developed that can be integrated with DMHP, based on the findings of the SENIOR (Support Systems Evaluation of Neuropsychiatric Illness in Old age) project

*ANM* auxiliary nurse midwife, *ASHA* accredited social health activist, *AUD* alcohol use disorder, *DMHP* District Mental Health Programme, *ECHO* Extension for Community Healthcare Outcomes, *JRF* junior research fellow, *LSEM* life skills education module, *NIMHANS* National Institute of Mental Health and Neuro-Sciences, *NMHP* National Mental Health Programme, *SRF* senior research fellow, multilingual modules (Marathi, Hindi, Kannada, Orissa)

^a^Includes accepted, published or presented at conferences

Several PIs were new to proposal writing and estimating budgets. They were regularly informed by the NCU, with additional support from mentors. At the first Grantathon meeting, PIs presented and defended their protocols tentatively, especially where multiple PIs and sites were involved, demonstrating hesitation and apprehension about sharing their ideas. Some had never presented in research meetings. When the mentors realized that several PIs had proposed similar projects, they suggested that they collaborate on a single project with multiple sites, rather than run individual projects. This approach had the advantages of increasing sample size, enabling within-study comparisons of populations with varying characteristics and initiating the development of a collaborative research community across various Indian states. Though PIs were not initially enthusiastic, as review meetings progressed, senior mentors noted the increasing synergy between PIs of multicentric projects as well as their confidence when defending their work, preparing presentations and administering their projects. Regular communications from the NCU via email and WhatsApp encouraged open communication. The monthly and as-needed phone calls and virtual meetings between PIs and the NCU further enhanced collaboration.

### Barriers and challenges

Though PIs successfully implemented their research projects, several challenges emerged. Some projects involved multiple stakeholders (e.g. state government, healthcare workers), creating additional implementation complications. In some cases, sites had to be moved due to unexpected circumstances, and one project was delayed due to floods. In other cases, PIs needed copyright permissions to use interventional tools, entailing delays. In multicentric studies in particular, project implementation was delayed due to procedural hurdles. The most frequently cited challenges were a lack of timely administrative support and time required for clinical responsibilities, which all PIs had to address regardless of their locations. This was particularly salient for PIs who were clinicians in nonacademic settings where the concept of protected time for research activities was not part of institutional culture. Thus, many PIs completed their research work outside office hours, in addition to their routine job responsibilities. The Grantathon specifically trained junior and mid-level faculty and nongovernmental organizations. Several PIs and their institutions were unaware of rules and regulations governing implementation research with human participants. PIs received in-principle approvals from their institutions’ authorities for receiving funds and for conducting research, but some institutions had no mechanisms to receive research funds. Overall, these barriers affected the timelines of research projects yet offered important lessons learned for future work.

Unsurprisingly, the COVID-19 pandemic affected the implementation of the Grantathon projects. Nationwide, several hospitals and clinics closed down, and prospective research participants could not be contacted due to lockdowns. To tackle these exigencies, mentors became adept at developing and seeking approval for appropriate alternative approaches. Following approval, PIs used telephonic or virtual follow-ups to reduce study drop-out and enhance recruitment and follow-up wherever possible. At some sites, these models were also used to educate the participants about COVID-19 infection [12]. PIs reported that activities conducted under this model helped to reduce the stigma about mental illness and were well accepted by the participants. Participants were largely comfortable with telephonic follow-ups, and these approaches likely reduced drop-out. One site introduced incentives for the accredited social health activist (ASHA) field workers who had continued their follow-ups of seriously mentally ill patients during the pandemic. Adding incentives for number of patients reached or other project-specific outcomes is a novel refinement to this approach to care. PIs also identified emerging issues, such as COVID-19 vaccine hesitancy among pregnant women [13]. In response, the PIs submitted a new research proposal to the ICMR, which was funded.

## Discussion

The Grantathon model represents a unique approach for improving mental health research capacity in India. This model was used to initiate basic training in intervention research through a 5-day intensive hands-on workshop and to facilitate collaboration, international networking, long-term monitoring, and the development of research capacity and soft skills related to project administration. It is interesting to note that evaluation feedback from Grantathon trainees who participated in distance versus in-person training revealed no significant differences in terms of overall value of training or suggested areas for improvement. However, it will be important to monitor the strength and sustainability of collaborative relationships over time. It is likely that stronger research networks were built via in-person workshops than through those conducted virtually. Active support from the ICMR through the i-MANN database was integral to continued success. Senior ICMR scientists also provided mentoring and administrative support. Periodic, regular training of PIs helped them to update their knowledge and skills and sustain collaborations. Emphasizing presentations and publication helped PIs to disseminate their work. To our knowledge, this project is the first to provide incentive-based mental healthcare in the context of the ASHA model. It is thus a partnership model for mental health research to address challenges related to mental health service implementation in LMICs and potentially in high-income countries.

Our model enabled coordinated, mentored research activities and provided an interdisciplinary approach to address complex questions related to cultural adaptation, effectiveness, dissemination and implementation. During 4 years of extensive monitoring and mentoring, PIs reported the value of their mentors in improving their research skills. The Grantathon helped PIs conduct research in challenging environments, supported their individual learning and helped them address organizational and structural roadblocks. The NCU, with its supportive and hands-on role, complemented the mentorship. Several departments and institutions were involved across multicentric studies; these newly established interdisciplinary relationships created a framework for interdisciplinary research that will yield benefits for future collaborative and integrative research.

Interventional studies are difficult to initiate and sustain, as they require long-term training and sustainable funding [14]. PIs may prefer to undertake short-term, cross-sectional or survey studies [15]. This preference may be due to the lack of access to or hesitation to apply for funding in a competitive environment and inadequate budget management skills [16, 17]. ICMR funding for the Grantathon research projects—although for the same amount as for general grants—could maximize impact, given that funding was coupled with mentoring and support from more experienced researchers. This model boosted PIs’ confidence to undertake interventional studies in complex and challenging settings.

Grover et al. pointed out that Indian researchers struggle to publish their findings despite having well-conducted studies [15]. PIs participating in our model successfully published and disseminated their findings in various national and international conferences, even winning awards. The coordinated research activities helped support all aspects of research (e.g. analysing data, writing manuscripts) and provided ideas and encouragement for the publications.

PIs implemented newly developed mental health interventions in real-world settings to address various components of the NMHP, helping them understand the complexity of this work with a focus on system-level uptake and long-term sustainability. Most studies had to overcome significant human and material resource limitations and find innovative ways to leverage existing systems of care through strategies such as task sharing, wherein non-mental health specialists were trained to deliver basic psychotherapeutic interventions with expert supervision. Also, most of the manuals that were developed are in multiple Indian languages and ready for use by other researchers. Our model also supported bidirectional collaboration, including shared authorship in peer-reviewed journals, and provided unique opportunities for PIs to present their findings in various conferences, including the National Institutes of Health/Fogarty International Center Global Brain Network Meeting.

Although assessing the full impact of the Grantathon model is beyond the scope of our evaluation, it is also important to consider less tangible outcomes and their impact on Indian mental health research. PIs published collateral work, became increasingly participatory in group meetings, and used this opportunity as a springboard to apply for other courses and fellowships. We previously conducted a network analysis of publications coauthored by mentors and trainees participating in the Grantathon and our other training programmes and demonstrated that examining coauthorship is a feasible method for documenting research collaboration and measuring impact [18]. Future evaluation work can explore this and other ways of demonstrating the extent to which these PIs become leaders in Indian mental health research.

With this cohort of mental healthcare providers from across India who have now worked with one another over a 3-year period, combined with the structure and analytical power of the i-MANN database, the NMHP now has a well-established cadre of practitioners with much-needed experience in testing creative and feasible models of mental health interventions in real-world settings. The PIs are now ready to share lessons learned with other researchers. The success of this approach will require commitment from the grantee institutions to provide continued funds and protected research time for clinician-scientists leading the projects, including coverage for clinical responsibilities.

The success of the Grantathon model has many implications for the training of PIs. It can be used to develop the curriculum or course for a mentor to implement psychiatric research or to develop unique networks of funding agencies, mentors and research institutes in LMICs. Grantathon-developed interventions may improve access to mental health treatment in many LMICs and thus could help address the dearth of evidence-based mental health research and services. As these projects were implemented in real settings and found useful during the COVID-19 pandemic, these models are sustainable [13]. Further, the mutual and practical learning among PIs in areas of common interest can percolate to their students, other healthcare employees and their patients. Our approach emphasized interdependency, bidirectional knowledge generation and transfer among mentors and PIs across the United States and India. This rare experience paired public health specialists and mental health workers to find practical solutions.

Our study has several limitations. We have not systematically assessed barriers and facilitators for these projects nor calculated the cost-effectiveness of our model. In future studies, the impediments and facilitators of research outputs should be identified systematically using pre/post, mixed-methods designs.

## Conclusion

A Grantathon-based partnership/mentorship model for research training successfully enabled 12 research studies and launched research careers for 24 trainee PIs. The Grantathon facilitated relationships among PIs and mentors and helped to promote and support their career growth. In addition, the model helped develop sustainable and scalable interventions for NMHP, generated substantial data, and spurred the development of a national mental health database. This model is a valuable approach for training future generations of mental health researchers, especially in LMICs where resources are tenuous. Key ingredients for its success include funding agencies willing to entertain novel ideas, dedicated mentors willing to commit time and leadership, a flexible database structure, a dedicated coordinating unit and new investigators willing to learn and engage in collaborative learning to build research capacity.

## Supplementary Information

**Additional file 1: Table S1.** Brief details of new research grants awarded to principal investigators.

## Data Availability

Not applicable.
