# Supplementary material for: A mentored hands-on training model for scaling up implementation and intervention research in India: “connecting the dots”
Source: Health Res Policy Syst. 2023 May 2;21:33. doi: 10.1186/s12961-023-00980-0 (PMC10153774; doi:10.1186/s12961-023-00980-0)
Supplement: Supplementary file 1 — Additional file 1: Table S1. Brief details of new research grants awarded to principal investigators. [file 12961_2023_980_MOESM1_ESM.docx]

**Additional file 1: Table S1.** Brief details of new research grants awarded to Principal Investigators

| **Title of project** | **Source of funding** | **Duration** |
| --- | --- | --- |
| Impact of COVID-19 restriction on children with disability: Satabdi Chakraborty | Indian Council of Medical Research, New Delhi | 2 Years |
| A pilot project to evaluate efficacy of Yoga intervention for stress, anxiety and depression among health care professionals working in frontline COVID-19 tertiary care hospital of New Delhi: Ram Pratap Beniwal | Ministry of Department of Science and Technology (DST), Govt of India | 1 Year, 3 Months |
| A correlative study to evaluate the association between speech and facial parameters in person with depression using artificial intelligence (AI): Ram Pratap Beniwal | DBT-BIRAC-Bill Gates Foundation (Insilico Pvt Ltd ) | 1 Year |
| Establishing the relationship of depression with behavioral markers from digital phenotyping in Indian Context: Ram Pratap Beniwal | Mannki Health Science Pvt Ltd | 1 Year |
| Development of a Brief Psychological Intervention for Anxiety symptoms among Perinatal Women: A Multi-Centric Randomized Controlled Trial from India: BIND-P group | Indian Council of Medical Research, New Delhi | 2 Years |
| Effectiveness of an intervention to address COVID-19 vaccine hesitancy among pregnant and lactating women: a multi-centric randomized controlled trial from India” : BINMD-P group | Indian Council of Medical Research, New Delhi | 1 Year |
| Primary Prevention Interventions for Substance -use and Suicide Prevention Among Youth in Kashmir: Development, Administration and Evaluation | Indian Council of Medical Research, New Delhi | 3 Years |
| Multi-centric randomized controlled trial to evaluate the efficacy of telephone based psychosocial intervention on future suicide risk in suicide attempter | Indian Council of Medical Research, New Delhi | 3 years 6 months |
